# NATURE AND CHALLENGES OF TRANSNATIONAL FAMILY CAREGIVING: FINDINGS FROM A SYSTEMATIC LITERATURE REVIEW

**DOI:** 10.1093/geroni/igae098.2418

**Published:** 2024-12-31

**Authors:** Mohammad Hossain, Natalie Pope, Regina George

**Affiliations:** University of Kentucky, Lexington, Kentucky, United States; University of Kentucky, Lexington, Kentucky, United States; University of Alabama, Tuscaloosa, Alabama, United States

## Abstract

This study explored the nature and challenges of transnational family caregiving via a systematic literature review. Increasing international migration and global aging have resulted in growing instances of transnational family caregiving, which involves providing care for older adults across national borders. However, little is known about the nature and challenges of such caregiving. Following PRISMA guidelines, a structured literature search was conducted using targeted search terms (e.g., transnational*, cross-border*, caregiv*, burden*) in 10 electronic databases (e.g., AgeLine, MEDLINE, CINAHL). Predefined eligibility criteria included empirical papers: (i) published in peer-reviewed English language journals, (ii) focused on the nature and/or challenges of transnational caregiving, and (iii) focused on caregiving for older adults. After removing duplicates, screening the titles and abstracts, and reviewing remaining full-texts, nine articles were deemed eligible for inclusion. Studies showed that transnational caregiving involves provision of financial and emotional support, arranging care, mailing gifts and letters, making care decisions, and communicating via phone/video calls. Financial strain came from expenses in the host and support countries, including traveling back to the home country. Transnational caregivers experience guilt, worry, and distress related to the unpredictability of the care situation and their inability to be physically present. Strict immigration policies, inadequate employer support, and limited institutional care in home countries also present challenges. Despite limitations of research in this area (e.g., qualitative studies; small, non-random samples), this review highlights the nature and challenges experienced by transnational caregivers. Further empirical research is needed to deepen our understanding of this unique subpopulation of caregivers.

